# Diagnostic value of circulating genetically abnormal cells to support computed tomography for benign and malignant pulmonary nodules

**DOI:** 10.1186/s12885-022-09472-w

**Published:** 2022-04-09

**Authors:** Han Yang, Hongjie Chen, Guorui Zhang, Hongyi Li, Ran Ni, Yali Yu, Yepeng Zhang, Yongjun Wu, Hong Liu

**Affiliations:** 1 Department of Respiratory and Critical Care Medicine, The First Affiliated Hospital of Zhengzhou University, Zhengzhou, 450052 Henan Province China; 2 General Intensive Care Unit, The First Affiliated Hospital of Zhengzhou University, Zhengzhou, 450052 Henan Province China; 3 Emergency Intensive Care Unit, The First Affiliated Hospital of Zhengzhou University, Zhengzhou, 450052 Henan Province China; 4 College of Public Health, Zhengzhou University, Zhengzhou, 450001 Henan Province China

**Keywords:** Circulating genetically abnormal cells (CAC), Pulmonary nodules, Lung cancer, Early diagnosis, Computed tomography (CT)

## Abstract

**Background:**

The accuracy of CT and tumour markers in screening lung cancer needs to be improved. Computer-aided diagnosis has been reported to effectively improve the diagnostic accuracy of imaging data, and recent studies have shown that circulating genetically abnormal cell (CAC) has the potential to become a novel marker of lung cancer. The purpose of this research is explore new ways of lung cancer screening.

**Methods:**

From May 2020 to April 2021, patients with pulmonary nodules who had received CAC examination within one week before surgery or biopsy at First Affiliated Hospital of Zhengzhou University were enrolled. CAC counts, CT scan images, serum tumour marker (CEA, CYFRA21–1, NSE) levels and demographic characteristics of the patients were collected for analysis. CT were uploaded to the Pulmonary Nodules Artificial Intelligence Diagnostic System (PNAIDS) to assess the malignancy probability of nodules. We compared diagnosis based on PNAIDS, CAC, Mayo Clinic Model, tumour markers alone and their combination. The combination models were built through logistic regression, and was compared through the area under (AUC) the ROC curve.

**Results:**

A total of 93 of 111 patients were included. The AUC of PNAIDS was 0.696, which increased to 0.847 when combined with CAC. The sensitivity (SE), specificity (SP), and positive (PPV) and negative (NPV) predictive values of the combined model were 61.0%, 94.1%, 94.7% and 58.2%, respectively. In addition, we evaluated the diagnostic value of CAC, which showed an AUC of 0.779, an SE of 76.3%, an SP of 64.7%, a PPV of 78.9%, and an NPV of 61.1%, higher than those of any single serum tumour marker and Mayo Clinic Model. The combination of PNAIDS and CAC exhibited significantly higher AUC values than the PNAIDS (*P* = 0.009) or the CAC (*P* = 0.047) indicator alone. However, including additional tumour markers did not significantly alter the performance of CAC and PNAIDS.

**Conclusions:**

CAC had a higher diagnostic value than traditional tumour markers in early-stage lung cancer and a supportive value for PNAIDS in the diagnosis of cancer based on lung nodules. The results of this study offer a new mode of screening for early-stage lung cancer using lung nodules.

**Supplementary Information:**

The online version contains supplementary material available at 10.1186/s12885-022-09472-w.

## Background

Lung cancer is the main contributor to cancer mortality globally [1, 2]. The main screening method for the early diagnosis of lung cancer is low-dose spiral CT (LDCT) when lung nodules are small in size, for which aspiration biopsy is not suitable. However, according to the report of the National Lung Screening Test (NLST), only 3.6% of lung nodules screened by LDCT are diagnosed as lung cancer [3], and this situation often causes overdiagnosis or a significant delay in the early diagnosis of lung cancer, and patients may lose the opportunity to receive timely treatment [4]. Moreover, due to differences in the experience and understanding of imaging readers, there remains a need for a method to assist in the analysis of CT results.

To date, there have been considerable efforts to improve the efficiency of diagnosis of lung cancer based on imaging, which includes computer-aided diagnosis (CAD) systems. Indeed, CAD systems can help in detecting lung nodules in LDCT and in determining the nature of nodules by extracting and analysing the imaging characteristics of nodules, including their size, shape, and density, among others [5]. A matched case–control study using NLST data found that the CAD image analysis method significantly improves diagnostic accuracy for lung nodules detected at low-dose CT [6]. Nevertheless, the imaging features of early-stage lung cancer are usually atypical, and it is still a challenge to use CAD alone to separate small malignant nodules from the majority of benign nodules. Furthermore, CAD lacks rigorous evidence to make explainable medical decisions because of the black-box-based inference process of deep learning [7]. Therefore, CAD cannot be applied for medical diagnosis and decision-making alone, yet the combination of multiple clinical indicators may help to improve diagnostic accuracy [7–9].

Besides a more reliable method to analyse and interpret CT results, biomarker tests from blood sample are also with great potential in lung cancer diagnosis. In addition to traditional tumour markers, noninvasive liquid biopsies, such as circulating free nucleic acids (RNA and DNA) and circulating tumour cells (CTCs), have been reported in recent years.

However, liquid biopsy has not yet been adopted in routine clinical practice owing to many limiting factors [10], and traditional tumour markers are limited because of their low sensitivity and false positives caused by infection or other factors [11]. Moreover, circulating tumour cells (CTCs) of lung cancer often display nonepithelial characteristics, and CTCs are difficult to detect through epithelial cell adhesion molecule (EpCAM)-dependent methods [12]. The recently proposed biomarker of circulating genetically abnormal cells (CACs) may solve this dilemma.

CACs are defined as peripheral blood mononuclear cells carrying mutations on chromosome 3 (3p22.1, 3q29) and chromosome 10 (10q22.3, CEP10); the detection of these cells are not EpCAM dependent and therefore overcome the limitation of CTCs detection [13]. Abnormalities at the above loci have been shown through comparative genomic hybridization analysis to commonly occur in lung cancer [14]. Katz et al. then confirmed genomic abnormalities in the sputum, tissue and blood of patients with non-small-cell lung cancer (NSCLC) [15–17]. Katz et al. also proved that CACs have auxiliary diagnostic value in different stages of lung cancer, with the latest research showing a sensitivity and specificity of 88.8% and 100%, respectively, for lung cancer diagnosis [17]. Therefore, CACs have great potential for diagnosing pulmonary nodules [18].

In this work, we retrospectively analysed data for patients with pulmonary nodules and attempted to identify a novel biomarker to support the ability of CT to differentiate malignant from benign pulmonary nodules. The objective of this study was to explore new ways of diagnosing pulmonary nodules by establishing new diagnostic models based on artificial intelligence-based CAD and comparing the diagnostic efficiency of different models.

## Methods

### Study design and patients

This was a retrospective study of patients with pulmonary nodules detected by CT at First Affiliated Hospital of Zhengzhou University; Totally, 111 patients were included from May 2020 to April 2021.

**Fig. 1:**
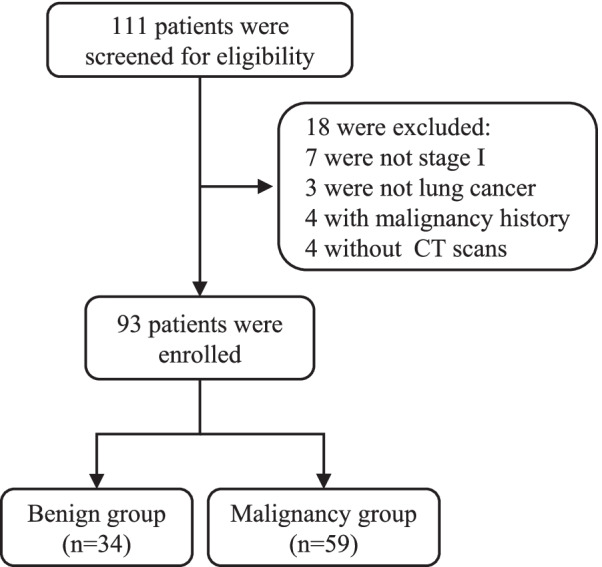
Flowchart of patients enrolled

The inclusion criteria for the study were as follows: (1) ≥ 18 years of age; (2) pulmonary nodule diameter no more than 30 mm (measured by CT scan), including single and multiple pulmonary nodules; (3) diagnosis histologically confirmed using nonsurgical biopsy (including fibre bronchoscope biopsy, computed tomography or ultrasonic-guided percutaneous transthoracic biopsy) or surgical resection; and (4) CAC tests performed within 1 week prior to surgery or biopsy. The exclusion criteria were as follows: (1) CT slice thickness greater than 2 mm; (2) a history of malignant tumours; (3) malignant nodules that were not classified as stage I based on the 8th edition of the American Joint Committee on Cancer (AJCC) staging system [19]; and (4) malignant nodules that were not primary malignant tumours of the lung. Ultimately, 93 patients were enrolled and divided into benign and malignancy groups based on histopathologic results (Fig. 1). Tumour pathology was classified according to the World Health Organization (WHO) classification standard of lung tumours (2015 edition) [20].

### Data collection

Clinical data for the patients were collected, including sex, age, smoking history and family history of malignant tumours. The results of preoperative serum tumour marker levels, including carcinoembryonic antigen (CEA), cytokeratin fragment 21–1 (CYFRA21–1) and neuron-specific enolase (NSE), for 66 patients were collected. The chest CT imaging data for the enrolled patients were separately exported, and the imaging features of nodules (including the diameter, type, location, counts, number and spiculation of nodules) were independently assessed by two senior physicians. When opinions differed, a consistent conclusion was reached through discussion with the third senior physician.

### Pulmonary Nodules Artificial Intelligence Diagnostic System (PNAIDS) based CAD

PNAIDS is an artificial intelligence-based CAD that applies machine learning technology and a deep convolutional neural network to realize 3D reconstruction and segmentation of nodules and predict the malignant probability of pulmonary nodules [21]. All chest CT scans were obtained during deep inspiration; the CT images were of no more than 5 mm of layer thickness and reconstructed with a slice thickness less than 2 mm. Imaging of the lung window was downloaded in DICOM format and uploaded to a cloud platform in the same format. The malignancy probability of each nodule was calculated. The highest malignancy probability value of all nodules was used for analysing patients with multiple nodules.

### CAC detection

**Fig. 2:**
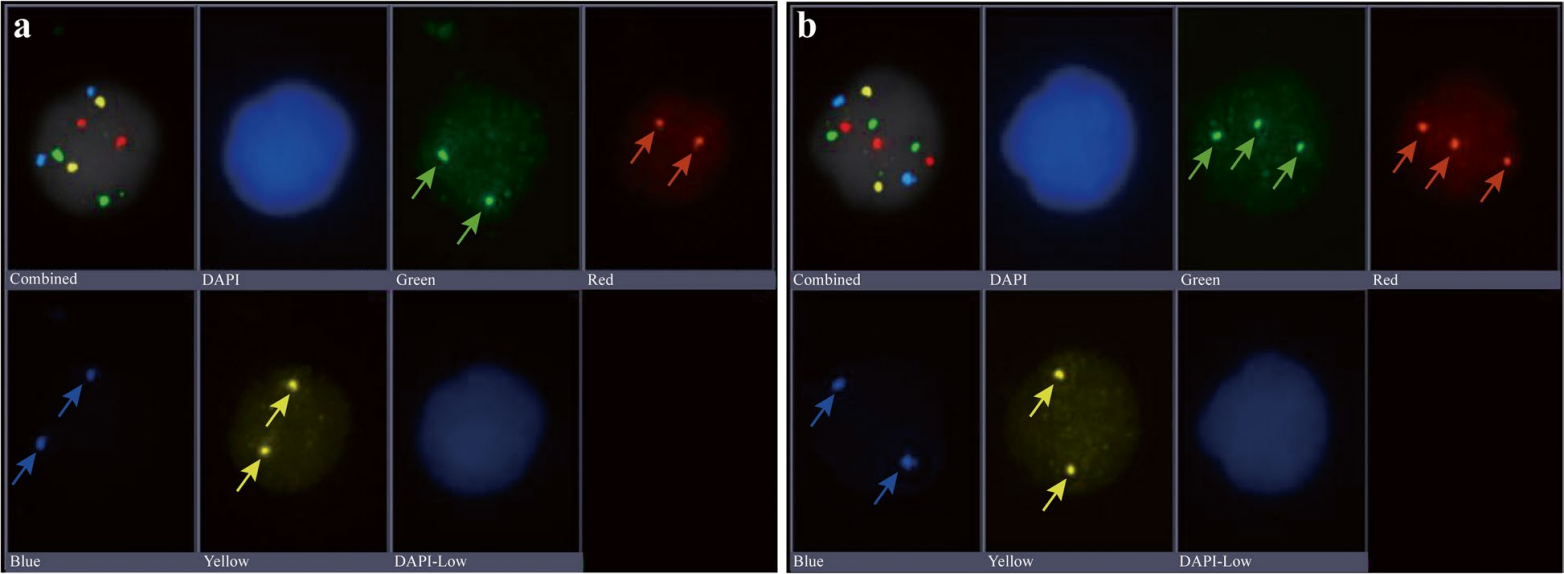
Interpretation of CAC test results **a)** the green and red probe which located in chromosome 3 have two signals (see green and red arrows in Fig. 2a), the blue and yellow probes located in chromosome 10 have two signals (see blue and yellow arrows in Fig. 2a), indicating normal cells. **b)** both the green and red probe which located in chromosome 3 have three signals (see green and red arrows in Fig. 2b), the blue and yellow probes located in chromosome 10 have two signals (see blue and yellow arrows in Fig. 2b), indicating that the cell has abnormal amplification on chromosome 3, which is CAC

Ten millilitres of peripheral venous blood was collected within one week before surgery or biopsy, blood samples were collected into an anticoagulation tube containing EDTA and fixed with cell preservation solution (including solution A containing phosphatase inhibitor and protease inhibitor and solution B containing formaldehyde) within 2 h. Peripheral blood mononuclear cells (PBMCs) were isolated by Ficoll-Hypaque density gradient centrifugation within 96 h. PBMCs were diluted to 40,000/100 μl, and a smear was prepared. Four-colour (3p22.1, 3q29 and 10q22.3, CEP10) fluorescence in situ hybridization was performed using a mononuclear cell chromosome abnormality detection kit (Zhuhai SanMed Biotech Inc.). The scanning, imaging and analysis procedures were automatically completed by a pathological section scanner (The Duet System, Allegro Plus, Bioview Ltd.). A total of 10,000 cells were randomly selected for a 15-layer cell scan, and the number of CACs was calculated. CACs were defined as cells exhibiting abnormal amplification at specific sites and at least three fluorescent signals at two or more specific probe sites (as presented in Fig. 2).

### Mayo Clinic model

The widely accepted Mayo Clinic model [22] was also performed to predict the malignant probability of nodules. The model expresses the malignancy probability as a function of six predictors: (1) probability of malignancy = *e*^x^ / (1 + *e*^x^); (2) *x* = -6.8272 + (0.0391 × age) + (0.7917 × smoking) + (1.3388 × cancer) + (0.1274 × nodule diameter) + (1.0407 × spiculation) + (0.7838 × upper lobe); (3) *e* is the natural logarithm; age is the patient's age (years), if the patient is a current or former smoker, smoking = 1 (otherwise = 0); if the patient has a history of extra-thoracic malignancy more than 5 years, cancer = 1 (otherwise = 0); the nodule diameter is the diameter of the nodule (mm); if there are burrs at the edge of the nodule, spiculation = 1 (otherwise = 0); if the nodules are located in the superior lobe, upper lobe = 1 (otherwise = 0).

### Statistical analysis

Statistical analyses were performed using SPSS 21.0. Quantitative variables are expressed as the mean ± standard deviation (*X* ± *S*) or median and quartiles [M(QL, QU)], and independent sample t-tests or Mann–Whitney U tests were applied. Categorical variables are expressed as n (%) and analysed using the *Chi-square* test or Fisher’s exact test. A receiver operating characteristic curve (ROC) and area under the curve (AUC), sensitivity (SE), specificity (SP), positive predictive value (PPV), negative predictive value (NPV) and Youden index was used to determine the cut-off value. To validate the robustness of the diagnostic model, logistic regression and Fisher discriminate analysis were both performed. The *Chi-square* test was applied for correlation analysis of classification variables. Two- sided *P* < 0.05 was considered significant. Correlation between numerical variables was analysed by calculating the Spearman rank correlation coefficient, with two-sided *P* < 0.01 considered significant. Boxplots, forest plots, and heatmaps were drawn in R (v4.0.10). DeLong’s test was applied to compare AUC between ROC curves (R package pROC).

## Results

### Patient characteristics

A total of 111 patients were initially screened in this study, among which 18 were excluded for different reasons (7 cases were not stage I, 3 were not primary lung cancer, 4 involved a malignancy history, 4 were without slice CT data) (Fig. 1). Ninety-three patients were ultimately included in the analysis, of which 59 (63.4%) were diagnosed with lung cancer and 34 (36.6%) with benign nodules. There were 39 males (41.9%) and 54 females (58.1%), with a mean age of 53.11 ± 10.74 years.

There were statistically significant differences in sex (*P* = 0.003), smoking history (*P* = 0.035), and type of nodules (*P* = 0.001), whereas no differences in age, family history of cancer, diameter of nodules, multiple nodules, upper lobe nodules, or burr signs were found between the benign group and the malignancy group. As none of the females had a history of smoking, a subgroup analysis of smoking history was performed, stratified by sex. Stratified analysis showed a nonsignificant difference in smoking history between the benign and malignancy groups in the male subgroup, with *Chi-square* test statistic of 0.300 (*P* = 0.584). The basic characteristics of the two groups are shown in Table 1.

**Table 1:** Demographic characteristics of patients

**Variables**	**Benign group (*n* = 34)**	**Malignancy group (*n* = 59)**	***x***^***2*** a^	***P***
**Gender, n (%)**			8.654	0.003
Male	21 (61.8%)	18 (30.5%)		
Female	13 (38.2%)	41 (69.5%)		
**Age, year**	51.27 ± 10.53	54.17 ± 10.81	1.260^b^	0.211
**Smoking history, n (%)**				
All patients			4.446	0.035
Yes	10 (29.4%)	7 (11.9%)		
No	24 (70.6%)	52 (88.1%)		
Male			0.300	0.584
Yes	10 (47.6%)	7 (38.9%)		
No	11 (52.4%)	11 (61.1%)		
Female			-	-
Yes	0	0		
No	13 (100%)	41 (100%)		
**Family history of cancer, n (%)**			0.431	0.512
Yes	7 (20.6%)	9 (15.3%)		
No	27 (79.4%)	50 (84.7%)		
**Nodule diameter (d), n (%)**			1.830^c^	0.419
5 mm < d ≤ 10 mm	17 (50.0%)	29 (49.2%)		
10 mm < d ≤ 20 mm	12 (35.3%)	26 (44.1%)		
20 mm < d ≤ 30 mm	5 (14.7%)	4 (6.8%)		
**Type of nodule, n (%)**			15.267	0.001
Solid nodule	22 (64.7%)	14 (23.7%)		
Mixed ground-glass nodule	5 (14.7%)	19 (32.2%)		
Pure ground-glass nodule	7 (20.6%)	26 (44.1%)		
**Solitary nodule, n (%)**			0.593	0.441
Yes	10 (29.4%)	22 (37.3%)		
No	24 (70.6%)	37 (62.7%)		
**Location of nodule, n (%)**			0.037	0.848
Upper lobe	22 (64.7%)	37 (62.7%)		
Non-upper lobe	12 (35.3%)	22 (37.3%)		
**Spiculation, n (%)**			0.555	0.456
Yes	25 (77.8%)	39 (55.2%)		
No	9 (22.2%)	20 (44.8%)		
**Histology, n (%)**			-	-
Benign tumour	2 (5.9%)	-		
Granuloma	9 (26.5%)	-		
Chronic inflammation	18 (52.9%)	-		
Other^d^	5 (14.7%)	-		
Adenocarcinoma	-	57 (96.6%)		
Other^e^	-	2 (3.4%)		

^a^ The statistic of the *Chi-square* test

^b^ The statistic of the t-test

^c^ The statistic of Fisher’s exact test

^d^ Including 2 organizing pneumonia, 3 fibrous tissue hyperplasia

^e^ Including 1 squamous cell carcinoma, 1 small cell lung cancer

### PNAIDS and CAC counts in patients

**Fig. 3:**
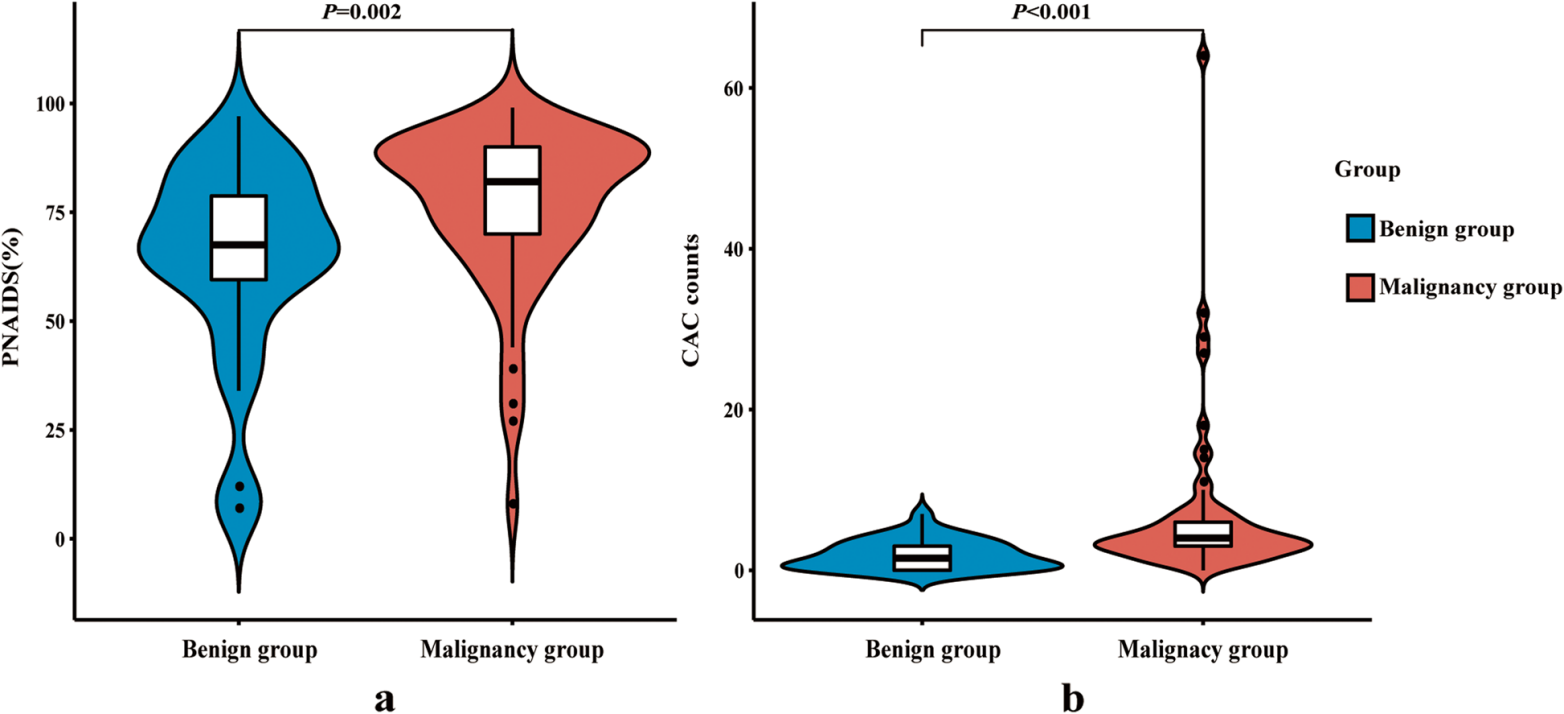
Distribution of **a)** PNAIDS and **b)** CAC counts in benign and malignancy group

There was no significant differences between the benign group and the malignancy group at the time before surgery or biopsy (3(1,5) days for benign group and 4(2,5) days for malignancy group; *P* = 0.393). The median CAC counts was 1.5(0, 3) in the benign group and 4 (3, 6) in the malignancy group; the median PNAIDS was 67.5% (59.5%, 78.8%) and 82.0% (70.0%, 90.0%), respectively. The distribution of CAC (*U* = 1562.5) and PNAIDS (*U* = 1396.5) between the benign and malignancy groups was statistically significant, at *P* < 0.001 and *P* = 0.002, respectively (Fig. 3).

### Diagnostic efficiency of different methods

**Fig. 4:**
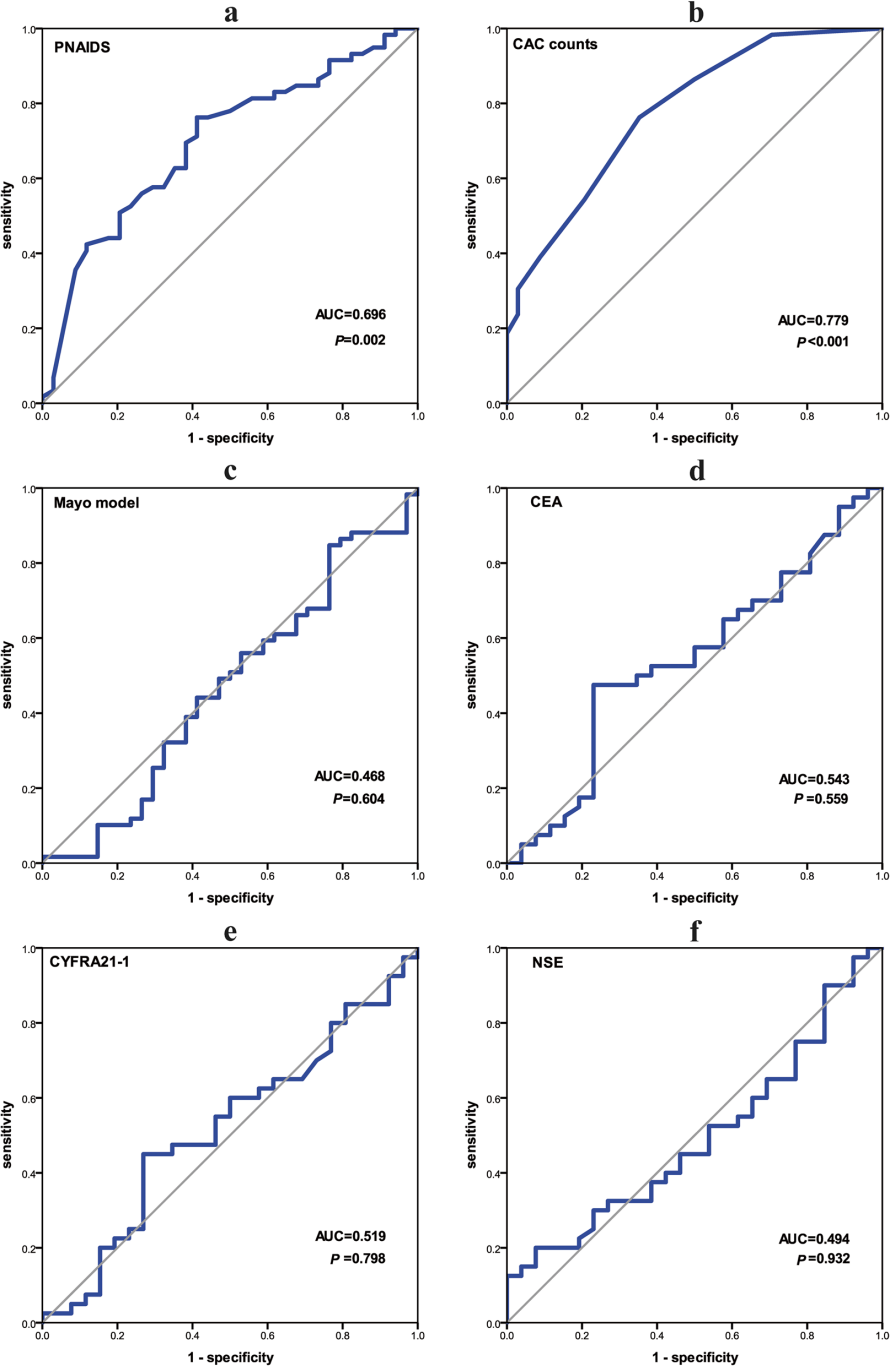
ROC of PNAIDS, CAC counts, Mayo model, CEA, CYFRA21–1 and NSE

**Fig. 5:**
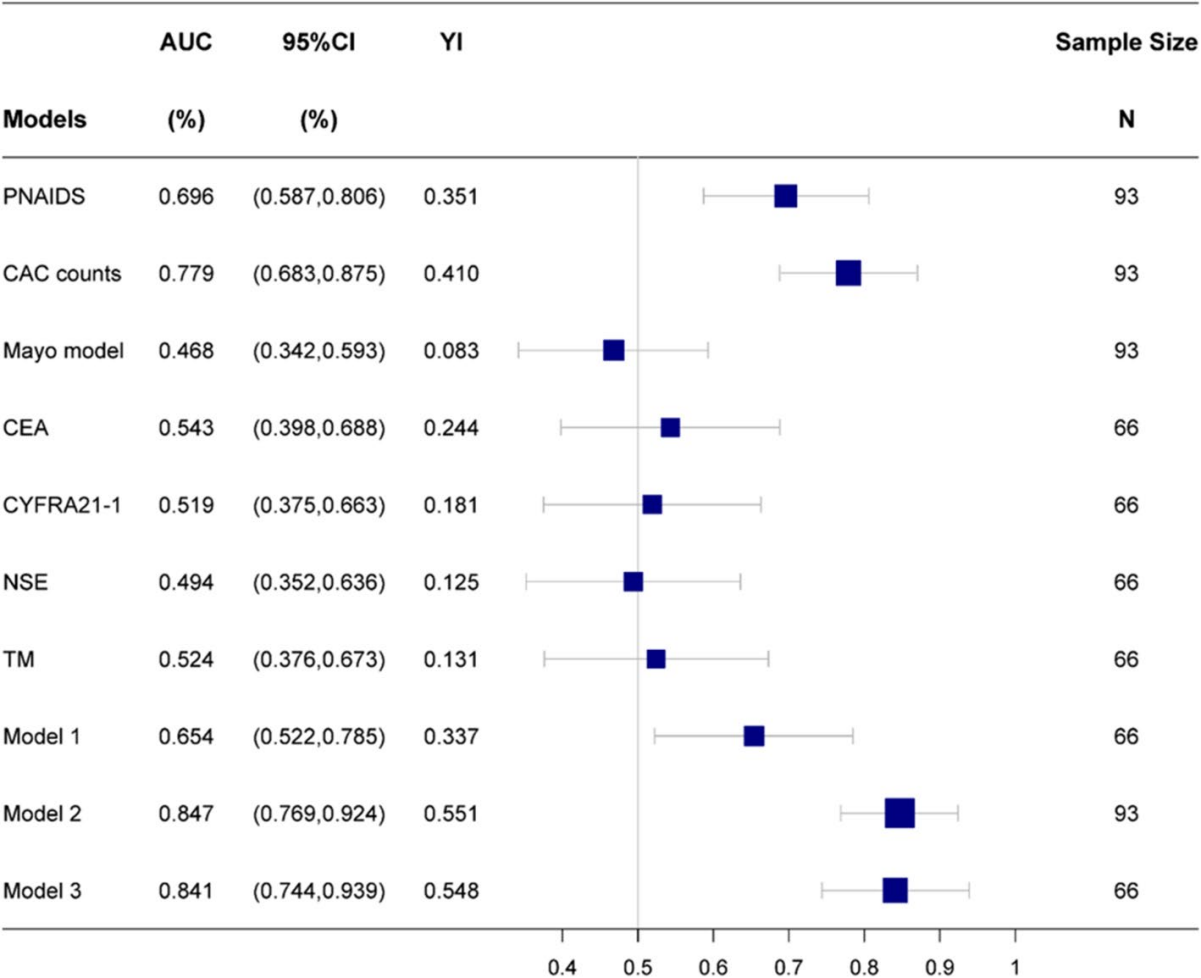
Forest plot of AUC: TM, the combination of CEA, CYFRA21–1 and NSE; Model 1, PNAIDS combined TM; Model 2, PNAIDS combined CAC; Model 3, PNAIDS combined CAC and TM

Based on PNAIDS, CAC counts, Mayo Clinic model, and tumour marker levels in the benign and malignancy groups, the ROCs were drawn (Fig. 4). The AUC, 95% confidence interval (CI), and Youden index of all these indicators are presented in Fig. 5. SE, SP, PPV and NPV are shown in Table 2. PNAIDS exhibited an AUC of 0.696, with a 95% CI of 0.587–0.806 (*P* = 0.002); the AUC of CAC counts was 0.779, and the 95% CI was 0.683–0.875 (*P* < 0.001).

**Table 2:** Comparison in different diagnostic methods

Models	AUC	95%CI		N	Youden index	SE	SP	PPV	NPV
PNAIDS	0.696	0.587-0.806		93	0.351	76.3%	58.8%	76.3%	58.8%
CAC counts	0.779	0.683-0.875		93	0.410	76.3%	64.7%	78.9%	61.1%
Mayo model	0.468	0.342-0.593		93	0.083	84.7%	23.5%	65.8%	47.1%
CEA	0.543	0.398-0.688		66	0.244	47.5%	76.9%	76.0%	48.8%
CYFRA21–1	0.519	0.375-0.663		66	0.181	45.0%	73.1%	72.0%	46.3%
NSE	0.494	0.352-0.636		66	0.125	12.5%	100.0%	100.0%	42.6%
TM	0.524	0.376-0.673		66	0.131	90.0%	23.1%	64.3%	60.0%
Model 1	0.654	0.522-0.785		66	0.337	37.5%	96.2%	93.8%	50.0%
Model 2	0.847	0.769-0.924		93	0.551	61.0%	94.1%	94.7%	58.2%
Model 3	0.841	0.744-0.939		66	0.548	62.5%	92.3%	92.6%	61.5%

*N* Number of samples, *SE* Sensitivity, *SP* Specificity, *PPV* Positive predictive value, *NPV* Negative predictive value; TM, the combination of CEA, CYFRA21–1 and NSE; Model 1, PNAIDS combined TM; Model 2, PNAIDS combined CAC; Model 3, PNAIDS combined CAC and TM

ROC curves demonstrated that 69.5% was the best cut-off value for PNAIDS, with the highest Youden index (0.351); the SE was 76.3% (45/59), the SP was 58.8% (20/34), the PPV was 76.3% (45/59), and the NPV was 58.8% (20/34). According to the ROC of CAC counts, the Youden index was highest when 2.5 CACs per 10,000 PBMCs was used as the cut-off. Cell count results was integer, so CAC counts ≥ 3 was chosen to discriminate benign and malignant nodules, with an SE of 76.3% (45/59), SP of 64.7% (22/34), PPV of 78.9% (45/57), and NPV of 61.1% (22/36).

### Correlation between indicators

**Fig. 6:**
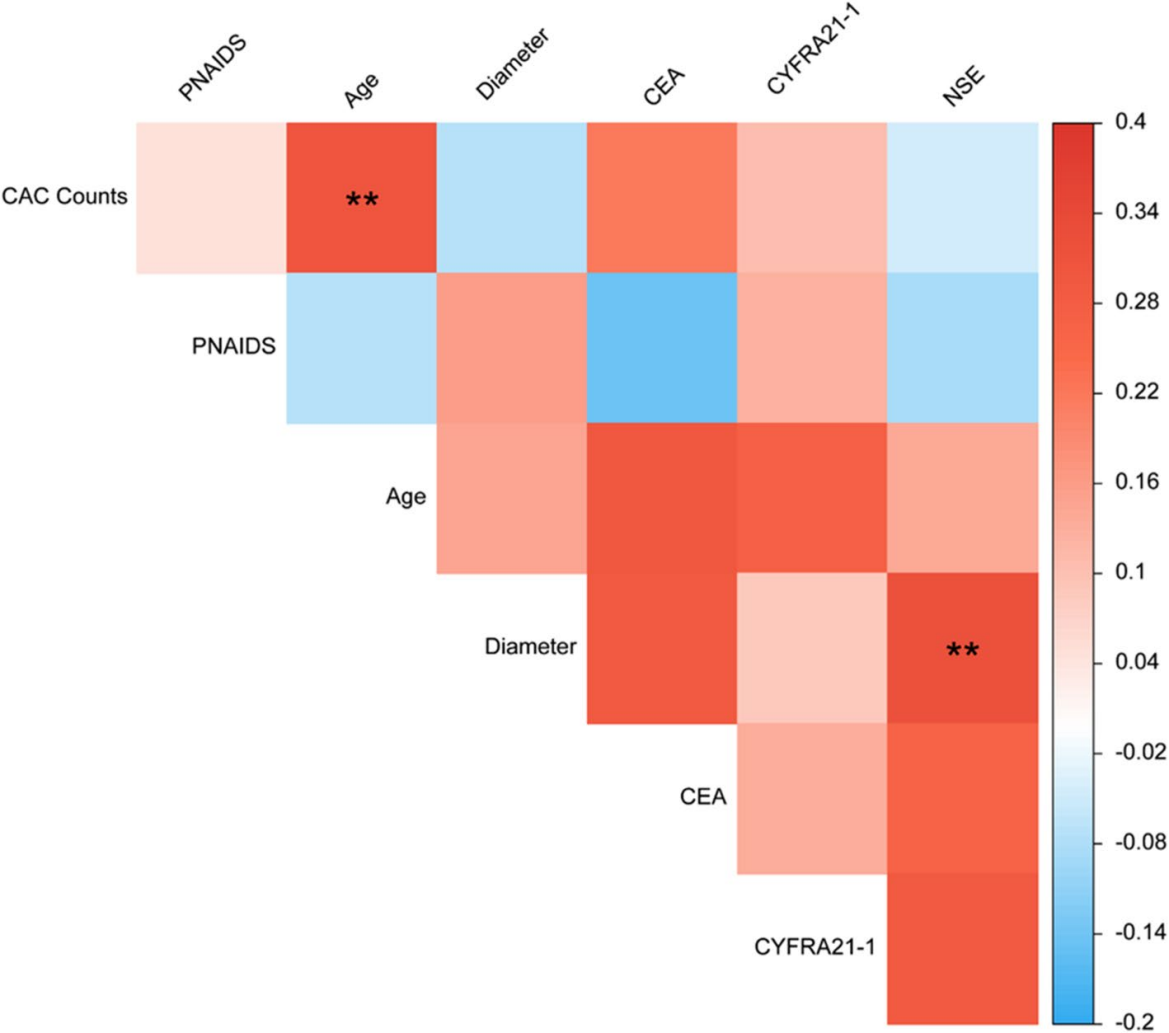
Correlation heatmap between PNAIDS, CAC counts, age, CEA, CYFRA21–1, NSE and nodule diameter (^**^P < 0.01)

Correlation analysis among CAC counts, PNAIDS, age, CEA, CYFRA21–1, NSE and nodule diameter showed a weak correlation between CAC counts and age (*r* = 0.311, *P* = 0.002), NSE and diameter of lung nodules (*r* = 0.323, *P* = 0.008). PNAIDS did not exhibited significant correlation with any of these indicators (Fig. 6). Notably, no significant correlation between PNAIDS and CAC was observed.

Numerical variables (PNAIDS and CAC counts) were converted to categorical variables according to cut-off values. Because PNAIDS was accurate to 2 decimal places, it was classified by whether it was less than 70.0%. In correlation analysis of PNAIDS, CAC counts and other categorical variables, PNAIDS correlated significantly with the type of nodule (*P* = 0.035), but no statistical significance with other indicators was detected. In addition, there was a nonsignificant correlation between CAC counts and all these categorical variables (Table 3).

**Table 3:** Correlation between CAC counts and PNAIDS with other Categorical variables

**Variables**	**CAC counts < 3 (*n* = 36)**	**CAC counts ≥ 3 (*n* = 57)**	***x***^***2*** a^	***P***	**PNAIDS < 70% (*n* = 34)**	**PNAIDS ≥ 70% (*n* = 59)**	***x***^***2*** a^	***P***
**Gender, n (%)**			0.152	0.697			1.431	0.232
Male	16 (44.4%)	23 (40.4%)			17 (50.0%)	22 (37.3%)		
Female	20 (55.6%)	34 (59.6%)			17 (50.0%)	37 (62.7%)		
**Family history of cancer, n (%)**			0.207	0.649			0.431	0.512
Yes	7 (19.4%)	9 (15.8%)			7 (20.6%)	9 (15.3%)		
No	29 (80.6%)	48 (84.2%)			27 (79.4%)	50 (84.7%)		
**Type of nodule, n (%)**			0.224	0.894			6.692	0.035
Solid nodule	15 (41.7%)	21 (36.8%)			19 (55.9%)	17 (28.8%)		
pure GGN	12 (33.3%)	21 (36.8%)			9 (26.5%)	24 (40.7%)		
mixed GGN	9 (25.0%)	15 (26.3%)			6 (17.6%)	18 (30.5%)		
**Solitary nodule, n (%)**			0.075	0.784			0.348	0.555
Yes	13 (36.1%)	19 (33.3%)			13 (38.2%)	19 (32.2%)		
No	23 (63.9%)	38 (66.7%)			21 (61.8%)	40 (67.8%)		
**Spiculation, n (%)**			0.317	0.573			2.494	0.114
Yes	26 (72.2%)	38 (66.7%)			20 (58.8%)	44 (74.6%)		
No	10 (27.8%)	19 (33.3%)			14 (41.2%)	15 (25.4%)		
**Location of nodule, n (%)**			1.953	0.162			2.547	0.110
Upper lobe	26 (72.2%)	33 (57.9%)			18 (52.9%)	41 (69.5%)		
Non-upper lobe	10 (27.8%)	24 (42.1%)			16 (47.1%)	18 (30.5%)		

^a^ The statistic of the *Chi-square* test

### Combination of different indicators for diagnosing lung nodules

**Fig. 7:**
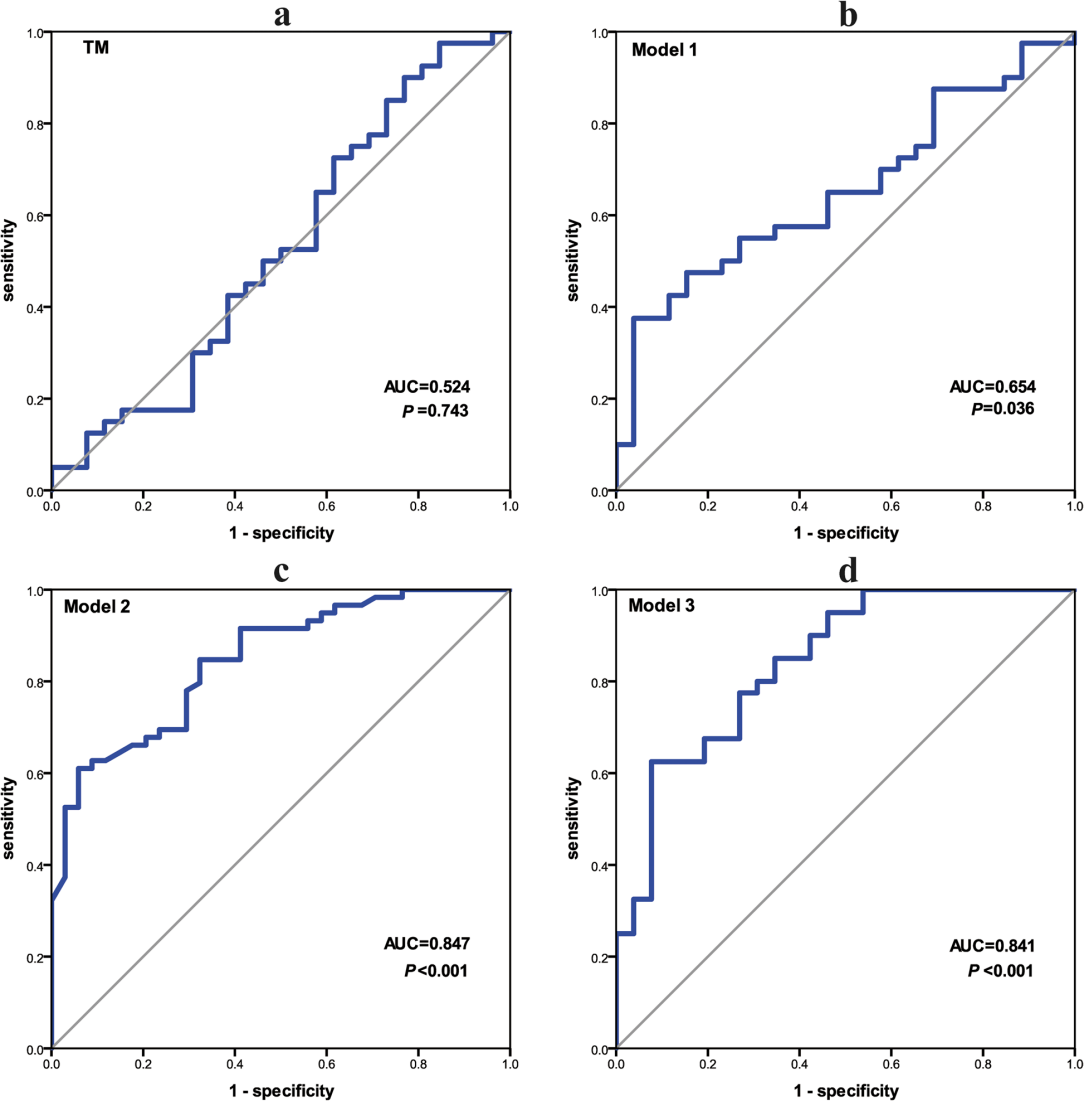
ROC of TM, Model 1, Model 2 and Model 3: TM, the combination of CEA, CYFRA21–1 and NSE; Model 1, PNAIDS combined TM; Model 2, PNAIDS combined CAC; Model 3, PNAIDS combined CAC and TM

As tumour markers tend to be used in combination in the clinic, a logistic regression model named TM was established using CEA, CYFRA21–1 and NSE, and its ROC curve used to diagnose lung nodules (Fig. 7). First, Model 1 was established by combining TM and PNAIDS. Second, PNAIDS and CAC counts were combined to build Model 2. To better apply CAC counts to the model, we transformed this marker into ln (CAC counts + 1), which was then applied to the logistic regression model. Similarly, the transformed data were used to build Model 3, which combined PNAIDS, CAC counts and TM. The ROCs of the three models are shown in Fig. 7; Fig. 5 and Table 2 provide the AUCs with 95% CI, SE, SP, PPV, NPV and Youden index values. Among all models, Model 2, based on the combination of PNAIDS and CAC counts, showed the highest diagnostic efficiency, at 0.847 (95% CI 0.769–0.924), with a significant improvement in AUC values compared with PNAIDS (*P* = 0.009) alone or CAC counts (*P* = 0.047) alone. Notably, Model 3 did not significantly improve the diagnostic efficiency compared with Model 2. Similar results were obtained in Fisher discriminant analysis (see Additional file 1). The formulas of logistic regression models are presented in Additional file 2.

**Fig. 8:**
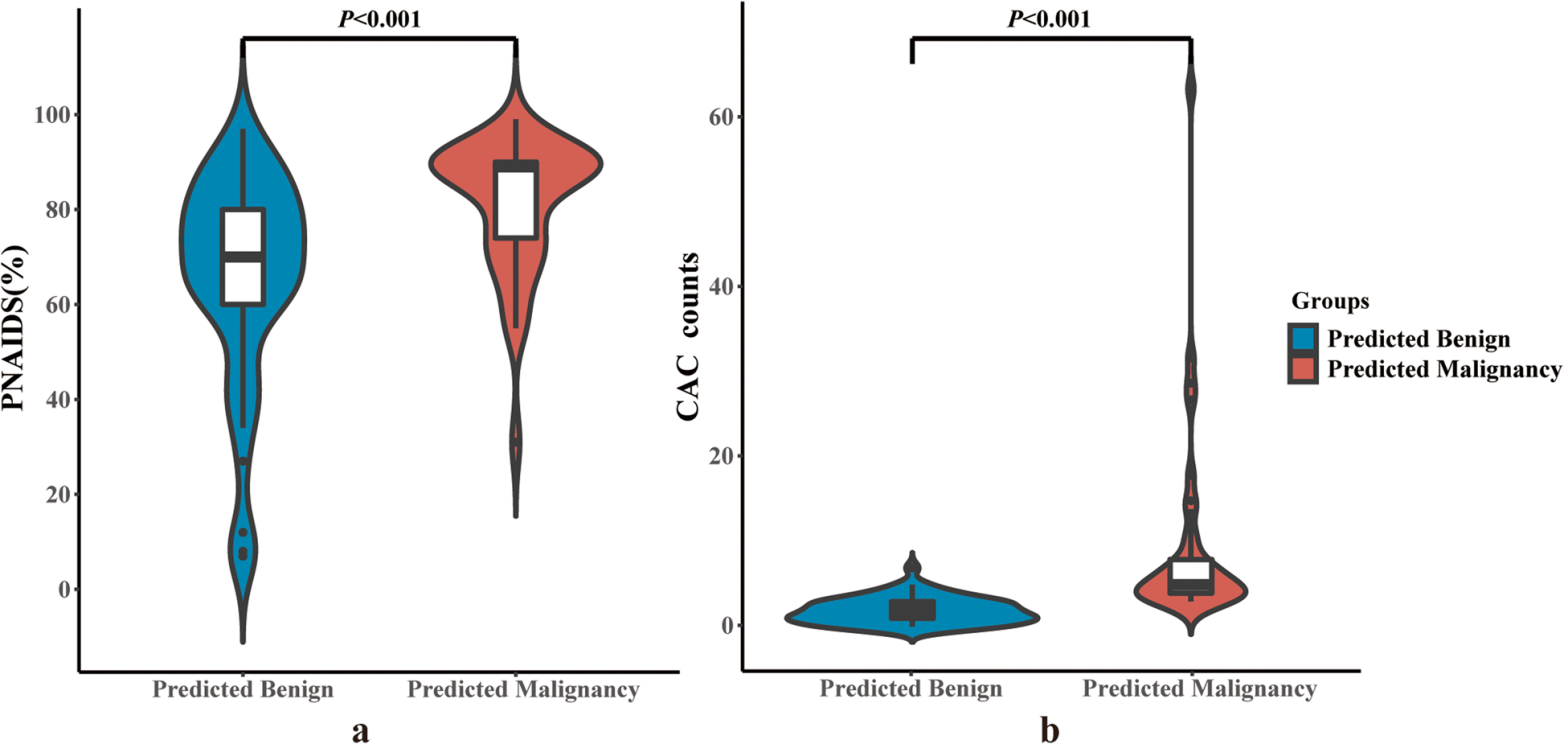
Distribution of **a)** PNAIDS and **b)** CAC counts in predicted benign and malignancy group(Model 2)

The best cut-off point was obtained according to the maximum Youden index in the ROC curve of Model 2 to divide patients into predicted benign and predicted malignancy groups. The distribution of CACs (*U* = 1936.0) and PNAIDS (*U* = 1550.5) between these groups was significantly different, with both at *P* < 0.001(Fig. 8).

## Discussion

In this study, the value of CT in discriminating lung cancer from small lung nodules was questioned, even with applying a current advanced artificial intelligence screening method which significantly increased the efficiency of diagnosis compared to the literature value of accuracy using CT [23]. Therefore, the traditional mode of small lung nodule diagnosis should be investigated. Previous works as well as this work suggest that CACs are an ideal candidate marker, as such a test only requires the simple process of blood collection and its accuracy has been reported in early lung cancer patients [18, 24, 25].

Despite the unsatisfactory result of the efficiency of CT alone, this approach still performed better than the Mayo Clinic model and tumour markers. As a common clinical imaging examination, CT has unquestionable value in the diagnosis of a variety of lung diseases [26]. A multicentre study involving 534 patients showed that PNAIDS had a higher diagnostic accuracy than the Mayo Clinic model and radiologists [21], consistent with our results. Several classic clinical indicators and imaging features are included in the Mayo Clinic model but show poor efficiency in distinguishing early lung cancer from benign pulmonary nodules. Thus, more specific imaging data may have higher diagnostic value than traditional imaging features.

Moreover, the diagnostic value of CACs in comparison with other traditional biomarkers has been confirmed using a cohort of patients with lung nodules, which is an independent validation to the work conducted by Ye et al. [24]. In the present study, the highest diagnostic efficiency was achieved when a CAC counts of 3 was chosen as the cut-off value, this result is similar to the study conducted by Qiu [25] and Ye [18] et al. Overall, CAC counts presented better diagnostic value than commonly used tumour markers (CEA, CYFRA21–1, and NSE), which agrees with the results of several studies reporting the advantages of CACs for the diagnosis of lung cancer [17, 18, 24, 25, 27], CACs have the potential to become a better novel diagnostic marker of lung cancer.

Biomarkers and imaging are often used in combination to improve diagnostic accuracy [8, 28], our results also showed that the efficiency has been greatly improved when CAC is combined with PNAIDS in the diagnosis of lung nodules. Correlation analysis further suggested that PNAIDS and CACs are independent of each other, which is consistent with the premise of the model that variables are independent. Interestingly, Model 2, which combined CAC counts and PNAIDS, displayed significantly higher diagnostic efficiency than CAC counts or PNAIDS alone. CAC counts and PNAIDS reflect the biogenetics and imaging features of patients, respectively. The 95% confidence intervals of the AUC of NSE, CYFRA21–1, CEA and the combined index TM all contained 0.5. However, Models 1 and 3, which further included TM, did not show improved diagnostic efficiency compared with PNAIDS or with PNAIDS combined with CAC. This result also suggests the limitation of the currently clinically used TM, that is, lack of sensitivity and specificity.

In addition, we analysed correlation between the indicators and demographic characteristics, which indicated a weak correlation between CAC counts and age. In the study by Liu [27], there was no relationship between a positive CAC result (CAC counts ≥ 1) and age (age ≥ 60), which is contrary to our finding. Liu et al. treated age and CAC counts as dichotomic variables, which may have led to poorer testing efficiency, whereas we directly analysed the correlation between age and CAC counts. The observed correlation may be due to genetic mutations that accumulate in cells with age, which also suggests that the age of the population may be a factor that needs to be controlled for or corrected in CAC detection. It should be noted that age is also a risk factor for NSCLC, further research is needed to explore whether there is a biological significance between CAC and age. The serum level of NSE had a significant correlation with nodular diameter, which can be explained by tumour burden [29, 30]. PNAIDS only showed a correlation with the type of nodule, suggesting the independence of imaging features and the value of imaging data for early screening of lung cancer.

Nevertheless, there are limitations in this study. First, as most malignant nodules screened by CT were adenocarcinoma, stratified analysis of different pathological types could not be applied to further explore the potential bias resulting from other pathological types of lung cancer. Second, smoking history was not common among the female patients, who comprised most cases; therefore, a larger sample size is required to assess the association between smoking and CAC counts or other indicators. Third, the sample size of this study was relatively small. Although the main statistical analysis yielded positive results, more studies with larger sample sizes are still needed to further confirm the practicability of our findings. Fourth, there is still scope to improve the diagnostic accuracy of PNAIDS, more data will be included in the future to train the PNAIDS model and construct a predictive model with higher accuracy. It is noteworthy that detection results of CACs can be obtained within 5 working days, quickly providing a more reliable auxiliary diagnostic basis when combined with PNAIDS, with a wide range of clinical application prospects. Our results indicate that this diagnostic model is promising for lung nodule diagnosis; with more data support in the future, it may be able to be extended worldwide.

## Conclusions

In conclusion, this work suggests that CACs, as a novel lung cancer biomarker from liquid biopsy, show higher diagnostic value than traditional tumour markers in early-stage lung cancer and a supportive value for CT scans in the diagnosis of cancer based on small lung nodules. The results of this study pave the way for further applications of CACs and offer a potential new mode for screening early-stage lung cancer using small lung nodules.

## Supplementary Information

**Additional file 1.**

**Additional file 2.**

## Data Availability

To preserve patient confidentiality the datasets generated for this study are not publicly available, but are available from the corresponding author on reasonable request.

## Abbreviations

AUC: Area under curve
AJCC: American Joint Committee on Cancer
CAC: Circulating genetically abnormal cells
CAD: Computer-aided diagnosis
CEA: Carcinoembryonic Antigen
CI: Confidence Interval
CT: Computed tomography
CYFRA21–1: Cytokeratin Fragment21–1
LDCT: Low-dose spiral CT
NLST: National Lung Screening Test
NPV: Negative predictive value
NSCLC: Non-small cell lung cancer
NSE: Neuron-Specific Enolase
PBMC: Peripheral blood mononuclear cells
PNAIDS: Pulmonary Nodules Artificial Intelligence Diagnostic System
PPV: Positive predictive value
ROC: Receiver operating characteristic
SE: Sensitivity
SP: Specificity
WHO: World Health Organization
