# Supplementary material for: Diagnostic value of circulating genetically abnormal cells to support computed tomography for benign and malignant pulmonary nodules
Source: BMC Cancer. 2022 Apr 9;22:382. doi: 10.1186/s12885-022-09472-w (PMC8994303; doi:10.1186/s12885-022-09472-w)
Supplement: Supplementary file 1 — Additional file 1. [file 12885_2022_9472_MOESM1_ESM.doc]

**Additional file 1**

**Fisher discriminant analysis**

The equations for Fisher's discriminant analysis:

TM’=-1.268-0.448×*CEA*-0.058×*CYFRA21-1*+0.186×*NSE*

Model 1’=-3.163+4.471×*PNAIDS*+0.433×*TM*

Model 2’=-3.812+2.941×*PNAIDS*+1.227×ln (*CAC counts*+1)

Model 3’=-3.398+2.028×*PNAIDS* +1.542×ln (*CAC counts*+1) +0.366×*TM*

(TM’, the combination of CEA, CYFRA21-1 and NSE; Model 1’, PNAIDS combined TM; Model 2’, PNAIDS combined CAC; Model 3’, PNAIDS combined CAC and TM’)


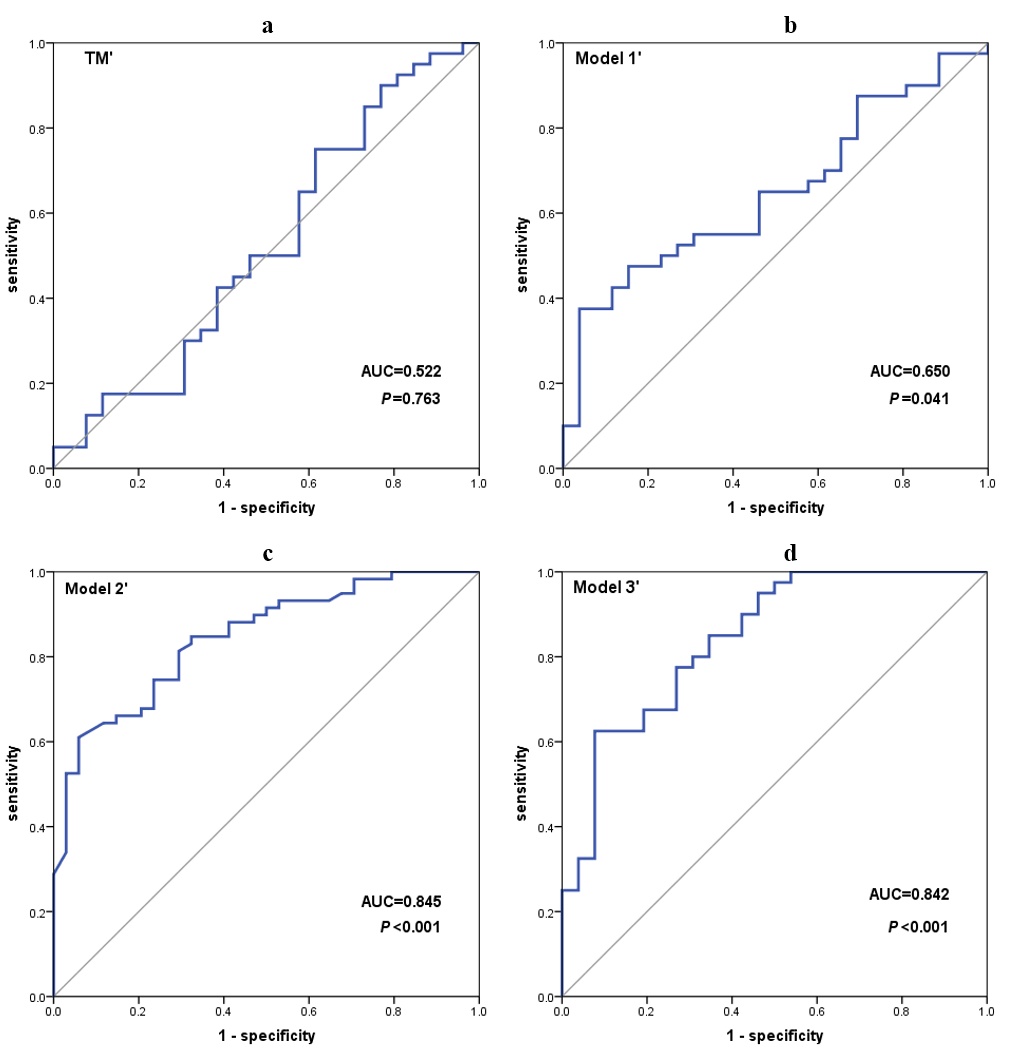


**Fig.A1** ROC of TM’, Model 1’, Model 2’ and Model 3’

**Table A1** Diagnostic efficacy of different models with discriminant analysis

|  | AUC | 95%CI | | N | YI | SE | SP | PPV | NPV |
| --- | --- | --- | --- | --- | --- | --- | --- | --- | --- |
| TM’ | 0.522 | 0.374 | 0.670 | 66 | 0.135 | 75.0% | 38.5% | 65.2% | 50.0% |
| Model 1’ | 0.650 | 0.518 | 0.782 | 66 | 0.337 | 37.5% | 96.2% | 93.8% | 50.0% |
| Model 2’ | 0.845 | 0.767 | 0.923 | 93 | 0.551 | 61.0% | 94.1% | 94.7% | 58.2% |
| Model 3’ | 0.842 | 0.745 | 0.939 | 66 | 0.548 | 62.5% | 92.3% | 92.6% | 61.5% |
