# Supplementary material for: Diagnostic value of circulating genetically abnormal cells to support computed tomography for benign and malignant pulmonary nodules
Source: BMC Cancer. 2022 Apr 9;22:382. doi: 10.1186/s12885-022-09472-w (PMC8994303; doi:10.1186/s12885-022-09472-w)
Supplement: Supplementary file 2 — Additional file 2. [file 12885_2022_9472_MOESM2_ESM.doc]

**Additional file 2**

**Formulas of logistic regression models:**

TM= *e*^x0^ / (1 + *e*^x0^)

Model 1= *e*^x1^ / (1 + *e*^x1^)

Model 2= *e*^x2^ / (1 + *e*^x2^)

Model 3= *e*^x3^ / (1 + *e*^x3^)

x_0_=0.155-0.094×*CEA*-0.013×*CYFRA21-1*+0.040×*NSE*

x_1_=-3.898+2.250×*PNAIDS*+4.547×*TM*

x_2_=-4.887 +4.068×*PNAIDS*+2.051×ln (*CAC counts*+1)

x_3_=-11.334+3.129×*PNAIDS* +2.483×ln (*CAC counts*+1) +11.042×*TM*
